# Supplementary figures and images for: Safety and feasibility of an in situ vaccination and immunomodulatory targeted radionuclide combination immuno-radiotherapy approach in a comparative (companion dog) setting
Source: PLoS One. 2021 Aug 12;16(8):e0255798. doi: 10.1371/journal.pone.0255798 (PMC8360580; doi:10.1371/journal.pone.0255798)

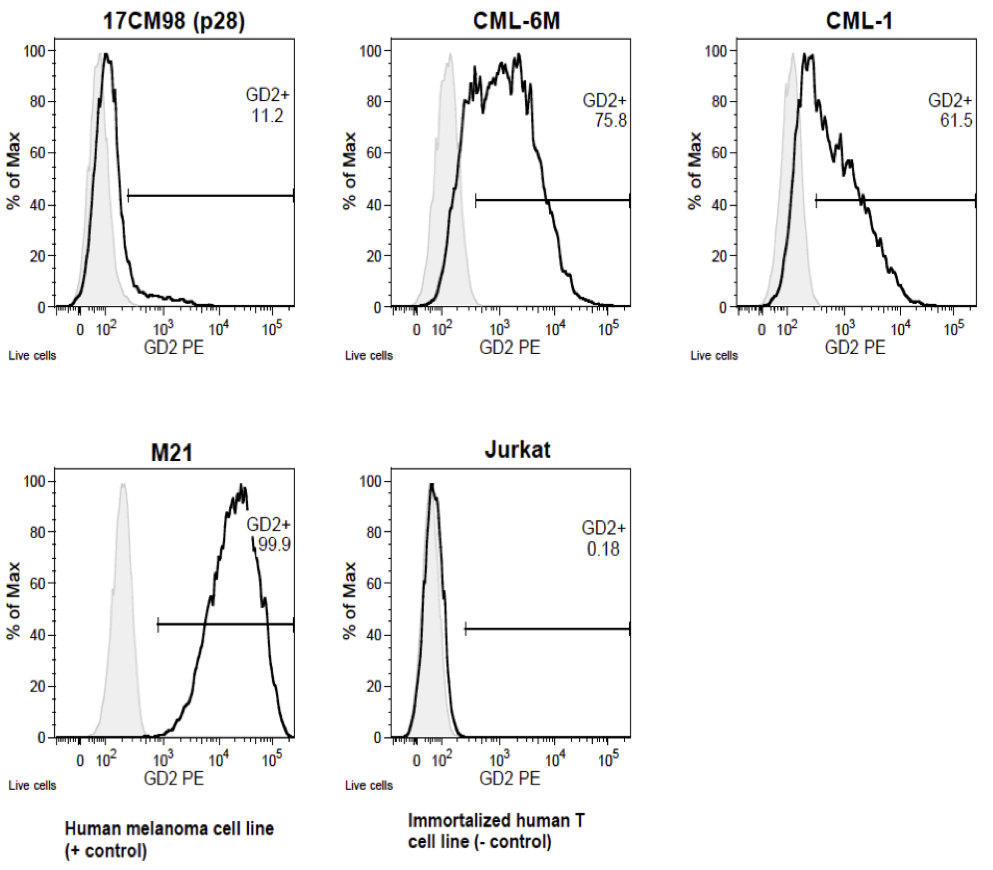

Supplement: S1 Fig — Thick black line indicates cells positively stained with anti-GD2-PE (clone 14.G2a, a murine IgG2a anti-GD2 mAb) and grey shaded areas indicate FMO control. Values indicate percent GD2+ cells. 17CM98, CML-6M and CML-1 are canine melanoma cell lines. M21 is a human GD2+ melanoma cell line used as a positive control. Jurkat is a human GD2- lymphoma cell line used as a negative control. (TIF) [file pone.0255798.s001.tif]

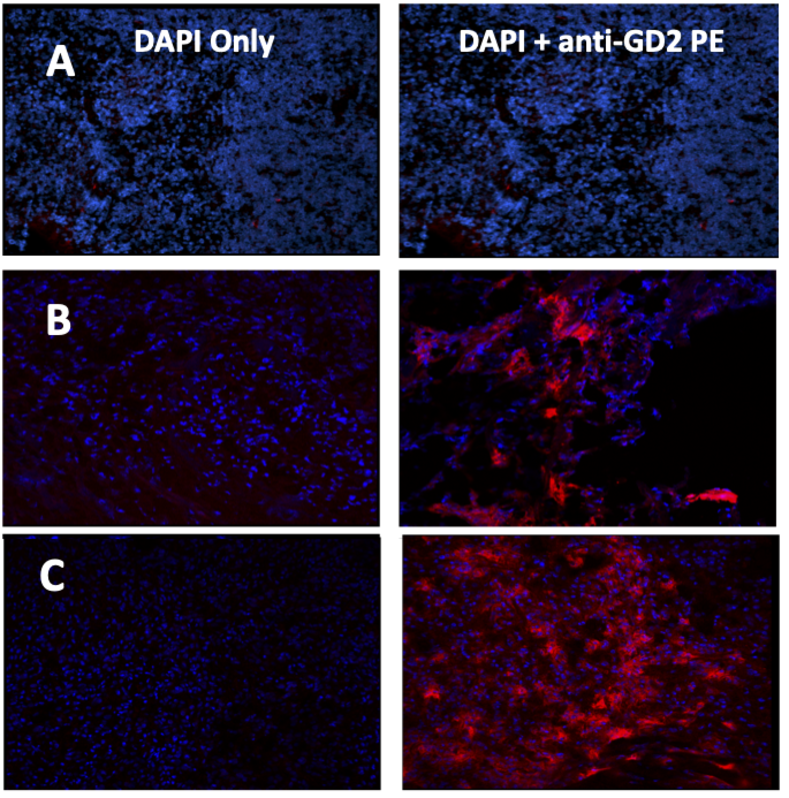

Supplement: S2 Fig — Frozen sections of canine tissues labeled with anti-GD2 mAb (clone 14.G2a, a murine IgG2a anti-GD2 mAb) conjugated to PE (red) and counter-stained with DAPI (blue). A, Canine spleen negative control; B, canine oral malignant melanoma; C, canine soft tissue sarcoma. (TIF) [file pone.0255798.s002.tif]

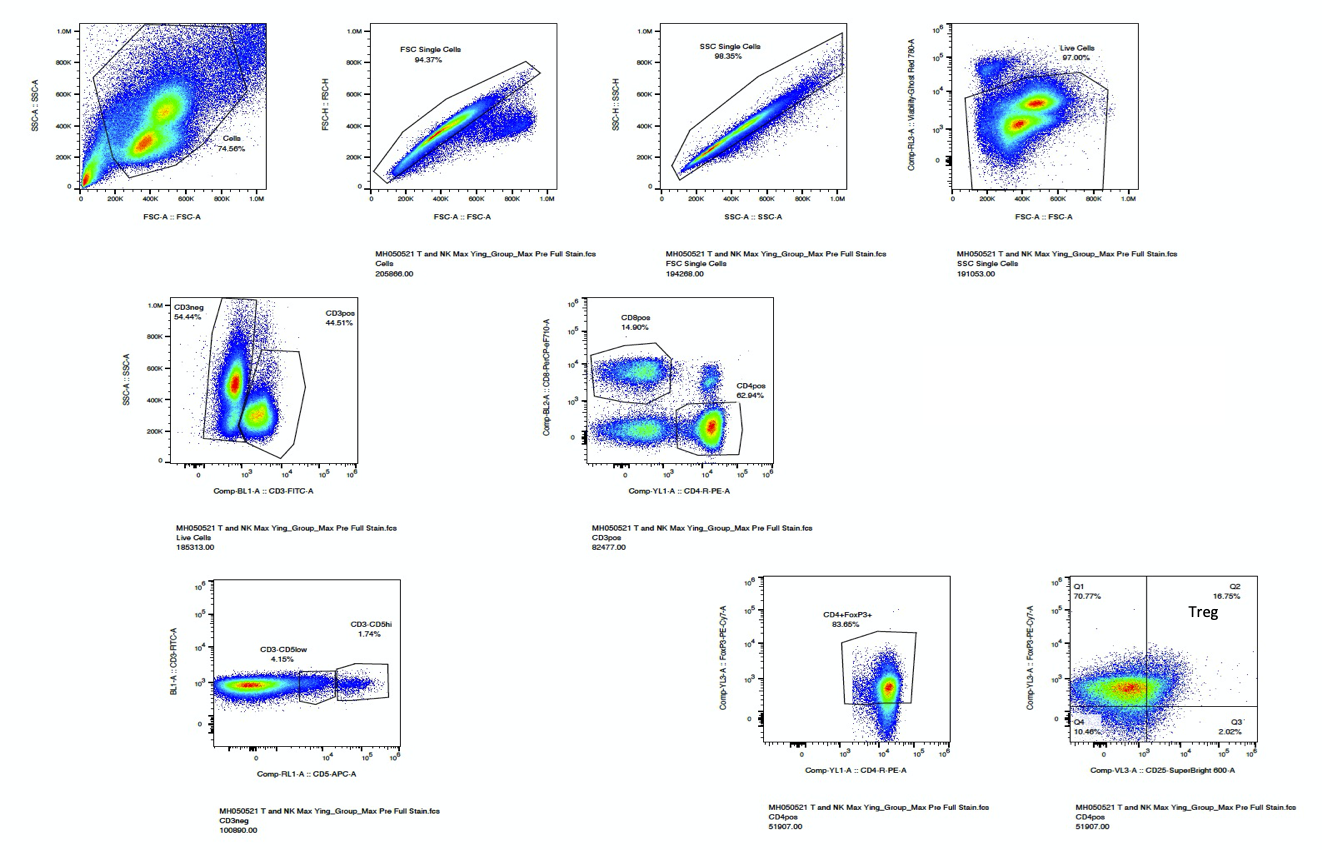

Supplement: S3 Fig — (TIF) [file pone.0255798.s003.tif]

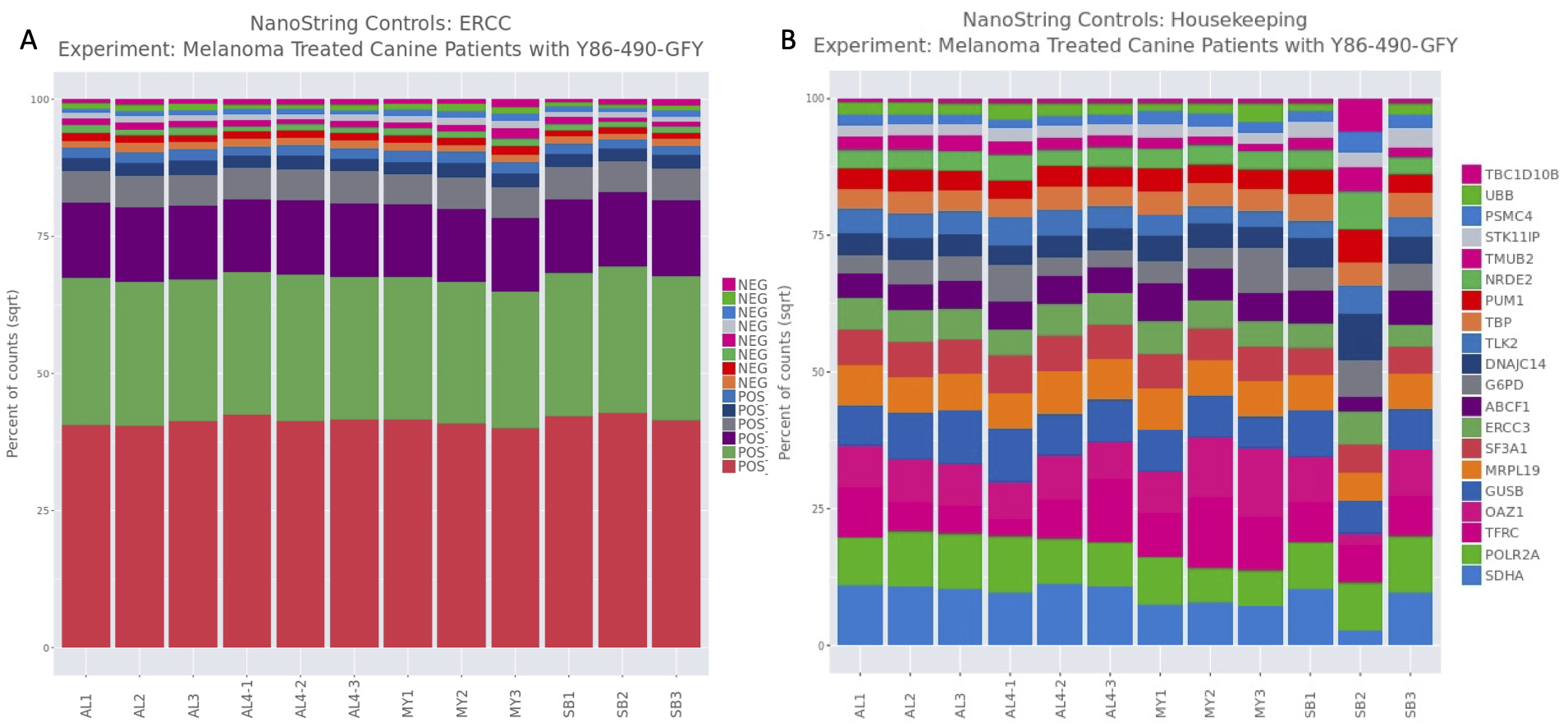

Supplement: S4 Fig — A, positive and negative controls. B, Housekeeping genes. The square root of the expression level is used to show the lower expression values. Note that specimen legend identifiers are coded and can be found in S3 Table. (TIF) [file pone.0255798.s004.tif]

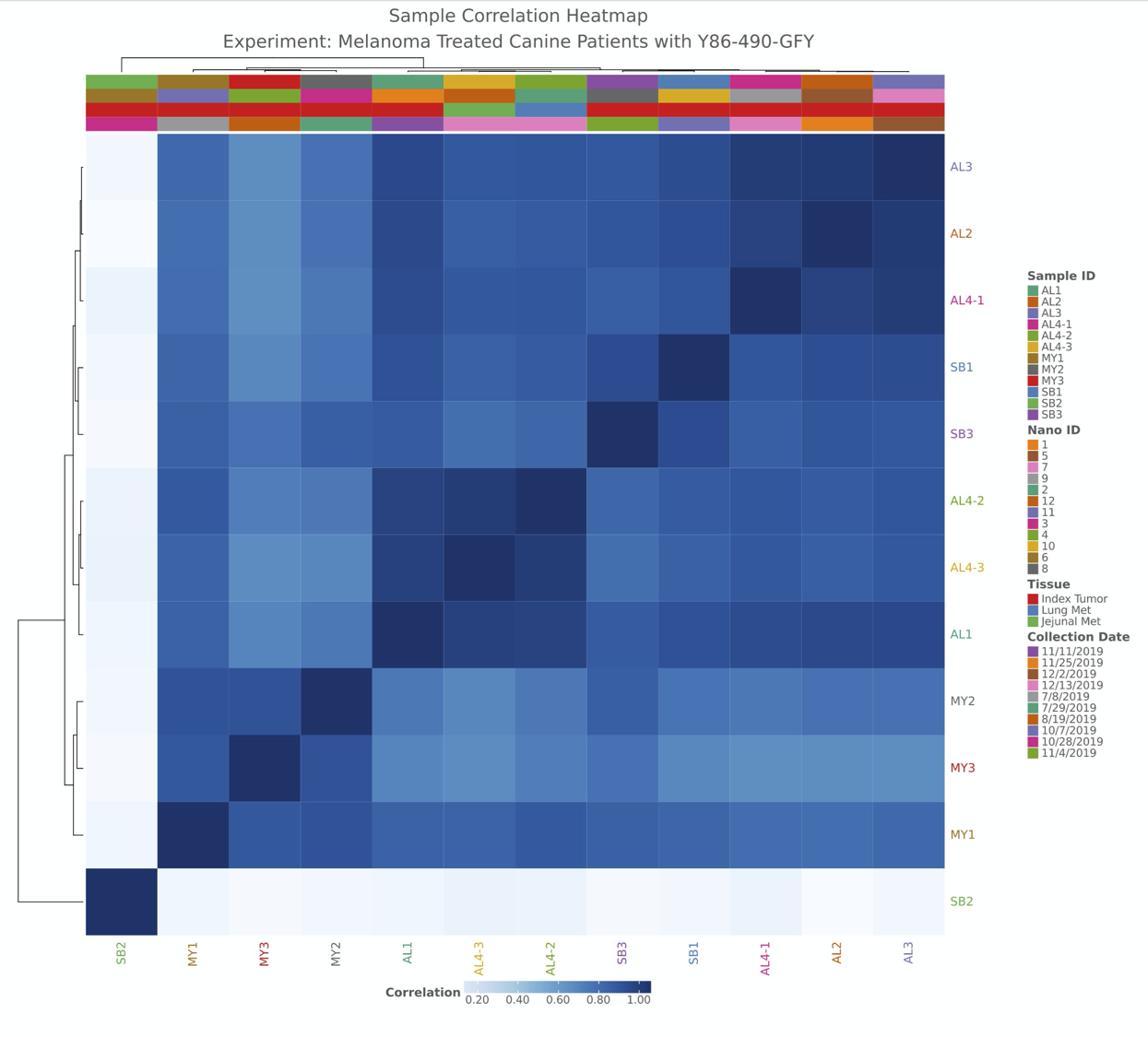

Supplement: S5 Fig — A sample correlation heatmap providing a graphical representation of data, in which the individual values contained in the matrix are represented as colors. In this case, the data matrix contains correlation values between samples, with the darkest blue representing the strongest correlation. The dendrogram annotation on the top axis provides information regarding the clustering of samples. Samples that are closely related (i.e., those in the same replicate group) are strongly correlated together in the plot and are the closest branches of the dendogram. Note that specimen legend identifiers are coded and can be found in S3 Table. (TIF) [file pone.0255798.s005.tif]

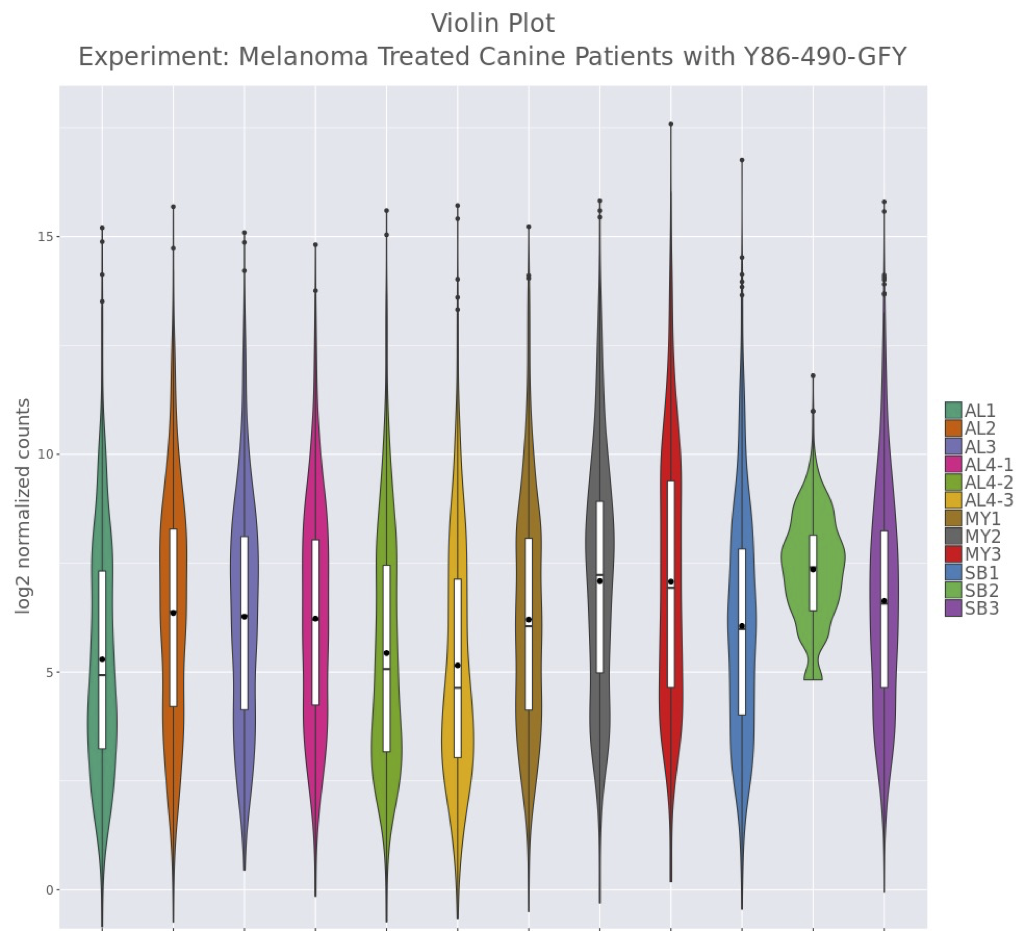

Supplement: S6 Fig — Note that specimen legend identifiers are coded and can be found in S3 Table. (TIF) [file pone.0255798.s006.tif]

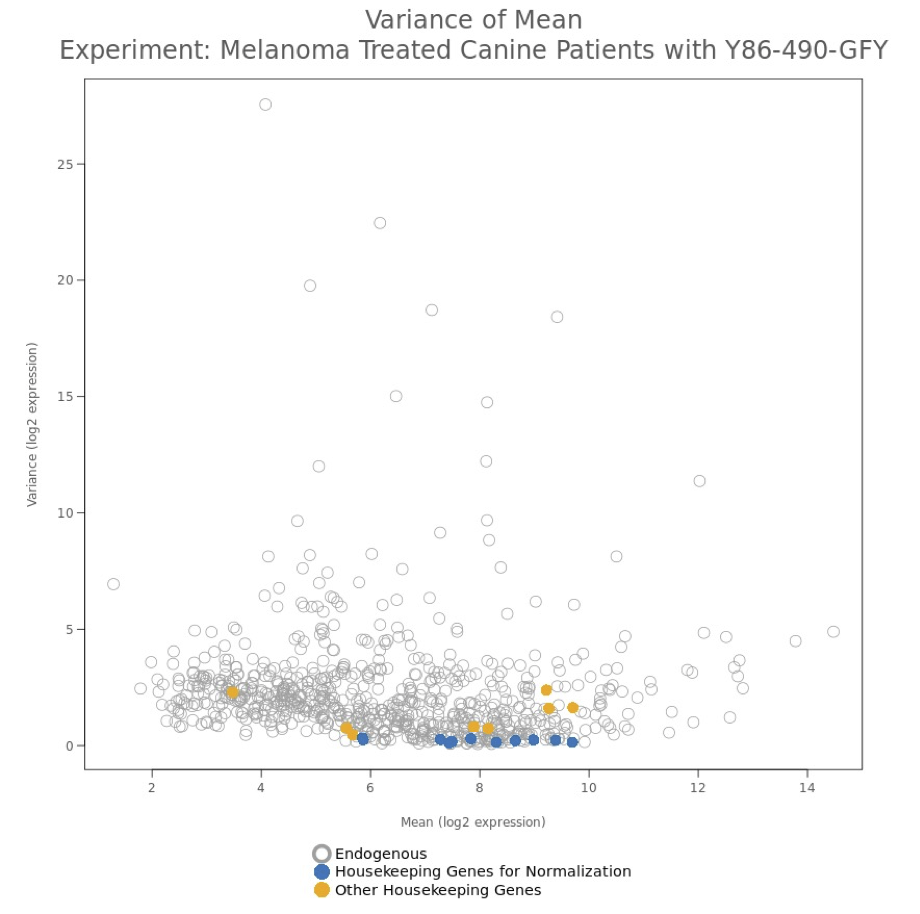

Supplement: S7 Fig — This plot maps the variance mean of the log2 expression of all targets and probes in the panel. Housekeeping probes are colored to indicate which ones were and were not used in normalization. (TIF) [file pone.0255798.s007.tif]
