# Supplementary material for: Safety and feasibility of an in situ vaccination and immunomodulatory targeted radionuclide combination immuno-radiotherapy approach in a comparative (companion dog) setting
Source: PLoS One. 2021 Aug 12;16(8):e0255798. doi: 10.1371/journal.pone.0255798 (PMC8360580; doi:10.1371/journal.pone.0255798)
Supplement: S1 Table — (DOCX) [file pone.0255798.s008.docx]

**S1 Table. Immunohistochemical reagents and parameters.**

| **Target** | **Primary Ab (Clone)** | **Dilution/incubation time** | **Secondary Ab** |
| --- | --- | --- | --- |
| **CD3** | DAKO (A0452) Rabbit polyclonal | 1:500/60 min | Anti-rabbit HRP Ventana #760-4311 |
| **CD4** | Origene (OTI10B5) Mouse monoclonal | 1:12000/16 min | Anti-mouse HRP Ventana #760-4310 |
| **CD8** | Abcam (ab4055)  Rabbit polyclonal | 1:200/60 min | Anti-rabbit HRP Ventana #760-4311 |
| **FOXP3** | Invitrogen (FJK-16s)  Rat monoclonal | 1:100/16 min | Anti-rat HRP Ventana #760-4457 |
