# Supplementary material for: Safety and feasibility of an in situ vaccination and immunomodulatory targeted radionuclide combination immuno-radiotherapy approach in a comparative (companion dog) setting
Source: PLoS One. 2021 Aug 12;16(8):e0255798. doi: 10.1371/journal.pone.0255798 (PMC8360580; doi:10.1371/journal.pone.0255798)
Supplement: S2 Table — APC, allophycocyanin; FITC, fluorescein; PE, phycoerythrin; PE-Cy7, PE-cyanine 7; PerCP-eFluor710, peridinin chlorophyll protein-eFluorTM710; SB60, Super Bright 600. (DOCX) [file pone.0255798.s009.docx]

**S2 Table. Flow cytometric reagents and parameters.**

| **Antigen/Marker** | **Conjugated**  **Fluorochrome** | **Antigen**  **Species** | **Antibody**  **Clone #** | **Manufacturer** | **Dilution** |
| --- | --- | --- | --- | --- | --- |
| CD3 | FITC | Canine | CA17.2A12 | Bio-Rad | 1:10 |
| CD4 | PE | Canine | YKIX302.9 | Bio-Rad | 1:10 |
| CD5 | APC | Canine | YKIX322.3 | Invitrogen | 1:10 |
| CD8 | PerCP-eFluor710 | Canine | YCATE55.9 | Invitrogen | 1:20 |
| CD25 | SB600 | Canine | P4A10 | Invitrogen | 1:10 |
| FoxP3 | PE-Cy7 | Rat | FJK-16s | Invitrogen | 1:25 |
