# Supplementary material for: Safety and feasibility of an in situ vaccination and immunomodulatory targeted radionuclide combination immuno-radiotherapy approach in a comparative (companion dog) setting
Source: PLoS One. 2021 Aug 12;16(8):e0255798. doi: 10.1371/journal.pone.0255798 (PMC8360580; doi:10.1371/journal.pone.0255798)
Supplement: S3 Table — (DOCX) [file pone.0255798.s010.docx]

**S3 Table. RNA quality of samples**.

| **Dog ID #** | **nCounter ID** | **Time Point** | **A260/280** | **A260/230** |
| --- | --- | --- | --- | --- |
| 2 | MY1 | Pretreatment | 2.14 | 2.25 |
|  | MY2 | Day 6 | 2.32 | 2.37 |
|  | MY3 | Day 13 | 2.15 | 2.31 |
| 3 | SB1 | Pretreatment | 2.09 | 1.94 |
|  | SB2 | Day 6 | 0.36 | 0.17 |
|  | SB3 | Day 13 | 2.11 | 2.17 |
| 4 | AL1 | Pretreatment | 2.18 | 2.35 |
|  | AL2 | Day 6 | 2.14 | 2.18 |
|  | AL3 | Day 13 | 2.23 | 2.40 |
|  | AL4-1 | Day 28 Index | 2.14 | 2.06 |
|  | AL4-2 | Day 28 Met1 | 2.13 | 2.24 |
|  | AL4-3 | Day 28 Met2 | 2.13 | 2.25 |
