# Supplementary material for: Safety and feasibility of an in situ vaccination and immunomodulatory targeted radionuclide combination immuno-radiotherapy approach in a comparative (companion dog) setting
Source: PLoS One. 2021 Aug 12;16(8):e0255798. doi: 10.1371/journal.pone.0255798 (PMC8360580; doi:10.1371/journal.pone.0255798)
Supplement: Attachment — Submitted filename: Response to Reviewers.docx [file pone.0255798.s011.docx]

June 21^th^, 2021

Editors, Plos One

RE: Response to Reviewers - manuscript PONE-D-21-01036.

Dear Editor:

We greatly appreciate the thoughtful comments and suggestions of the two reviewers and feel the changes and additions we have made serve only to make the manuscript better. In particular, while we had initially made a decision to exclude any treatment-related immunological assessments in this feasibility presentation, we have now added exploratory assessments of immunomodulatory changes in tumor gene expression levels (nCounter NanoString), tumor infiltrating lymphocyte subsets (nCounter cell type profiling, and IHC analysis) and peripheral circulating cell subset analysis (flow cytometric characterizations). These explorations, based on the first multi-modal immuno-radiotherapy cohort (n = 3) in our ongoing studies, provide preliminary evidence for immunomodulatory effects of our protocol which, when expanded in our ongoing studies should provide valuable data as to the mechanisms involved, allow for further modifications of protocol and characterize the utility of the surrogate system. We required an extension of the revision deadline to complete this exploration due to ongoing reagent availability and laboratory personnel density limits resulting from COVID restrictions at our institution.

**Authors Response to Reviewers Queries:**

**Reviewer #1:**

***General Comment: “…the study suffers in the lack of any immunological or tumor analysis data essential for being able to draw any conclusions on whether the regimen was having any immune or tumor effects (and importantly, if the regimen needs to be developed more as no DLT was observed on the single treatments especially given the extremely small sample size) and heterogeneity of the disease/breeds. There is a need for both local and systemic immune parameters (especially T and NK cells, serum cytokines etc) to be shown including during treatment and post-mortem when available. Extensive or in-depth assessments are not needed but given the preclinical and clinical use of the immunocytokine and the species divergence, it is essential to include immune parameters in the study.”***

We had initially presented only safety and feasibility data for this proof-of-concept first cohort while batching biospecimens (tumor/normal/PBMC, serum/plasma) samples for immune-analysis once ongoing expanded cohorts with increasing TRT doses were completed; however, we certainly agree that this was a deficiency in the initial manuscript. As COVID restrictions have relaxed somewhat in our laboratories, we now present, as requested, exploratory characterizations of immunomodulatory effects of our combined immune-radiotherapy approach in our first melanoma cohort (n=3). These include use of the nCounter Canine Immuno-Oncology (IO) gene expression panel that identified gene expression changes occurring one and 2 weeks after therapy as well as changes in tumor infiltrating lymphocyte populations by both nCounter cell type profiling capabilities and standard IHC analysis. Further we characterized peripheral circulating immune cell subset by flow cytometric characterizations at similar time points. These data are now presented in new figures and described in detail in methods and results under the subheadings ‘Exploratory assessment of immunomodulatory effects’ in addition to substantial text added to the discussion section pertaining to them.

While numbers are small these explorations reveal immunologic changes occurring over time resulting from our protocol. Confident and in-depth interpretation of these changes will ultimately require further interrogation of expanded cohorts and follow-up clinical data which are the subject of ongoing endeavors. Additionally, the effect to which each of the trimodal treatments (i.e., IT-IC, EBRT, and TRT) contributed to the observed immunomodulatory effects cannot be discerned from this data set. Planned interrogations on biospecimens collected from dogs receiving single-agent IT-IC, EBRT and IT-IC, and comparison to the trimodal (EBRT/TRT/IT-IC) groups (current and ongoing) will be necessary for such an assessment.

***Other points:***

1) ***the use of hu14.18-IL2 is at first confusing versus simply using IL2 as it is not expected that the anti-human GD2 mAb used will bind to canine GD2 at all. There needs to be much more background on this limitation and immunokine (how does the IL2 IU activity compare? does any cross-reactivity exist? Neutralizing responses (as totally human and therefore xenogeneic) by the patient? The discussion should include limitations on the canine model in these cases and draw also from the clinical data with this agent.***

Our group has previously worked with the hu14.18-IL2 immunocytokine in dogs and canine tissues. As presented in the original manuscript, reference 24 and supplemental figures 1 & 2 describes our groups work documenting GD2 expression in canine melanoma cell cultures and canine melanoma and sarcoma primary tumors by flow cytometry and immunofluorescence microscopy, respectively. Further, our groups work (ref 24) has documented that both the mouse 14.G2a monoclonal (used in our diagnostic flow and immunofluorescence microscopy assays) and the mouse-human chimeric 14.18 anti-GD2 mAb (used in our clinical fusion protein) enhanced lysis of canine melanoma cells by canine peripheral blood lymphocytes indicating their ability to direct ADCC by canine effector cells. Additionally, our group (ref 25) and several others have has also shown that human IL2 has significant immunologic effects in dogs and that the addition of human IL2 augments the ADCC potentiation of the 14.18 anti-GD2 mAb against canine melanoma cells (ref 24) as is the case in human melanoma. As GD2 is a disialoganglioside, it is not species specific, and the 14.18 anti-GD2 mAb can recognize mouse, human and canine melanoma. We have clarified this in the paragraph beginning on **line 107** of the revision (track-changes version). While there was no *a priori* requirement for GD2 expression in the proof-of-concept trials outlined in the manuscript, we plan on assessing this in batch at study completion. However, in the revised manuscript we did perform immunofluorescent microscopy on the index tumors in the 3 dogs with melanoma receiving the complete trimodal immuno-radiotherapy protocol (EBRT/TRT/IT-IC) and found 1 of 3 to express GD2. This is in line with human melanoma patients (ref 23) where 6 of 12 patents were GD2 positive. We have added this to the manuscript and addressed in the discussion. Regarding neutralizing responses (since it is a xenogeneic mAb), as requested, we have added new text to the discussion section (see the text at the end of the paragraph starting on **line 773**) as a limitation to recurring or multiple hu14.18-IL2 treatments and that caninized platforms would likely be necessary for repeated treatments in future studies.

2) ***there needs to be more information on the disease status and health of the client-owned dogs as well as information on prior treatments, if any as all impact toxicities.***

All dogs had measurable disease of at least 2 cm longest diameter (**line 180**). The tumor clinical stage for dogs in the EBRT/IT-IC combination protocol are listed on **line 473** in results. Further, the clinical stage for dogs in the EBRT/TRT/IT-IC combination protocol are listed in the manuscript in table 2 in results. Regarding general health, a statement that all dogs had to have a pretreatment constitutional clinical sign status of 0 or 1 (normal or asymptomatic/mild symptoms not requiring intervention) according to VCOG CTCAE v1.1 at entry was added to **line 176**. Regarding prior treatments, exclusion requirements (now listed in **Line 178**) dis-allowed prior radiation therapy or immunotherapy and required a minimum 2-week washout from previous chemotherapy. Prior surgery was allowable as long as post-surgical recurrence met the 2 cm minimum longest diameter cut-off.

**Reviewer #2:**

**1. Please include the number of dogs in the abstract**.

Added as requested.

**2. Prefer "dogs" as the noun and "canine" as the adjective**.

Corrected throughout.

3. **Please include the IACUC protocol number**.

Added to **line 166** in methods.

4. **The radiolabeling protocol is not described. Please briefly describe it and highlight any differences in protocol (if present) from the referenced article.**

The protocol was added as requested on lines **262-269.**

5. **What is the source and production methods of the Y-90 used?**

^90^Y was purchased from Perkin Elmer as ^90^YCl_3_ – a statement to this effect was added to methods (**line 261)**

**6.** **The term "strong partial" is difficult to quantitate. The result in the described dog was a partial response of 67% reduction**.

The term “strong” was deleted as requested.

**7. In the normal dog safety study, please include attribution with the hypo- and hyperglycemia as you do for the hematuria.**

As in the discussion, the following sentence was added to attribute the hypoglycemia (**line 494**). “Low grade fluctuations in blood glucose levels in these dogs was likely attributable to a lack of standardization in feeding schedule and time from blood collection to serum separation.”

**8. The tumor to bone marrow ratio calculation is difficult to understand in both the methods and the result. Looking at the values in Figure 3, it appears that only one dog actually had a primary tumor:bone marrow ratio > 2. This is explained somewhat further in the Discussion, but warrants clarification in the methods and results.**

We have clarified in the methods and results that the 2:1 tumor:bone marrow ratio requirement is for **metastatic** tumor:BM, not **primary** tumor:BM as the primary tumor receives 8Gy EBRT in the protocol and therefore is not considered relevant to the 2:1 ratio eligibility cut-off (**line 298**). Therefore, indeed 4 of 5 dogs met criteria and the one dog not meeting this criteria did not go on to receive TRT.

**9. Figures 1 and 2 were difficult to read in the version this reviewer received.**

We defer to the editors as fig 1 & 2 in our figure files are readable and we are not sure which versions were provided the reviewer.

**10. If the immune infiltrates or peripheral blood immune populations have been analyzed, the results should be included in this manuscript to round out preliminary evidence of effect along with feasibility.**

Please see response to reviewer #1’s general query. We have added gene expression changes (nCounter Canine Immuno-oncology [IO] gene expression panel) occurring one and 2 weeks after therapy as well as changes in tumor infiltrating lymphocyte populations by both nCounter cell type profiling capabilities and standard IHC analysis. Further we characterized changes in peripheral circulating immune cell subset by flow cytometric characterizations at similar time points. These data were added in addition to further methodology and discussion points pertaining to them.
